# Twist1 Suppresses Cementoblast Differentiation

**DOI:** 10.3390/dj6040057

**Published:** 2018-10-17

**Authors:** Jung-Sun Moon, Seong-Duk Kim, Hyun-Mi Ko, Young-Jun Kim, Sun-Hun Kim, Min-Seok Kim

**Affiliations:** Dental Science Research Institute, School of Dentistry, Chonnam National University, Gwangju 61186, Korea; u9598067@hanmail.net (J.-S.M.); 0186666@hanmail.net (S.-D.K.); gusalrh@hanmail.net (H.-M.K.); youngjun@jnu.ac.kr (Y.-J.K.); ksh@jnu.ac.kr (S.-H.K.)

**Keywords:** Twist1, cementoblast differentiation, OCCM-30

## Abstract

The transcription factor Twist1 is known to be closely associated with the formation of bone by mesenchymal stem cells and osteoblasts; however, the role of Twist1 in cementogenesis has not yet been determined. This study was undertaken to elucidate the roles of Twist1 in cementoblast differentiation by means of the gain- or loss-of-function method. We used alkaline phosphatase (ALP) and alizarin red S staining and quantitative real-time reverse transcriptase polymerase chain reaction (qRT-PCR) to determine whether the forced transient expression or knock-down of Twist1 in a mouse cementoblast cell line, OCCM-30, could affect cementogenic differentiation. Silencing Twist1 with small interference RNA (siRNA) enhanced the formation of mineralized tissue. The expression of several cementogenesis markers, such as bone sialoprotein (BSP), osteopontin (OPN), dentin matrix protein1 (DMP1), and dentin sialophosphoprotein (DSPP) mRNA, were upregulated. Transient Twist1 overexpression in OCCM-30 consistently suppressed mineralization capacity and downregulated the differentiation markers. These results suggest that the Twist1 transcription factor may play a role in regulating cementoblast differentiation.

## 1. Introduction

Although the cementum covering the root surface of a tooth is a calcified tooth constituent, it is classified as periodontal tissue because it has a common origin with the periodontal ligament and alveolar bone. Since it plays a critical role in the tooth-anchorage system, understanding the creation of cementum by cementoblasts is essential for regenerating functional teeth. Although the cementum shares several aspects with bone, including their biochemical composition, it differs in that it has no blood vessels and nerves histologically and has little remodeling capability, whereas bone has well-organized systems for regeneration by osteoblasts, osteocytes, and osteoclasts [1,2]. Both in vitro and in vivo studies have shown that noncollagenous proteins, such as alkaline phosphatase (ALP), bone sialoprotein (BSP), and osteocalcin (OCN), are synthesized by cementoblasts. In addition, gene expression of cementoblasts was found to be upregulated during differentiation induced by bone morphogenetic proteins [3,4,5,6]. However, little is known about detailed molecular mechanisms that underlie the process of cementoblast differentiation.

Twist-related protein 1 (Twist1), a member of the basic helix–loop–helix (bHLH) transcription factor family, plays a variety of roles in the development of mesodermally derived tissues, including bone, cartilage, muscle formation, and epithelial–mesenchymal transformation (EMT). Moreover, Twist1 is involved in controlling the determination of cell types and their differentiation [7,8,9,10,11]. Transcripts encoding this bHLH factor are expressed in mouse embryonic tissue, fetal cartilage, adult skeletal tissues, and osteoblast cell lines [8]. Twist1 affects the expression of genes for bone formation and regulates the osteogenic lineage by acting as a master switch for the initiation of osteoblast differentiation [10,11]. In addition, a wide range of signaling pathways involved in craniofacial development (e.g., teeth and palate) can functionally converge with bHLH transcription factors, including Twist1 [12]. However, the roles of Twist1 in cementogenesis have never been examined. In this study, we used alkaline phosphatase (ALP) and alizarin red S staining and quantitative real-time reverse transcriptase polymerase chain reaction (qRT-PCR) to determine whether the transient expression or knock-down of Twist1 in a mouse cementoblast cell line, OCCM-30, could alter cementoblast differentiation.

## 2. Materials and Methods

### 2.1. Cell Culture

The OCCM-30 established cementoblast cell line, which was derived from the dental root surface of transgenic mice containing SV 40 large T-antigen, was kindly provided by Dr. Martha J. Somerman (National Institutes of Health, Bethesda, MD, USA) [4]. OCCM-30 cells were cultured in growth media, Dulbecco’s Minimum Essential Medium (DMEM, Gibco BRL, Gaithersburg, MD, USA) supplemented with 10% fetal bovine serum (FBS, Gibco BRL), 100 U/mL of penicillin (Gibco BRL), and 100 μg/mL streptomycin (Gibco BRL). When confluency of cells reached 70 to 80%, they were used for subsequent experiments. For cementoblast differentiation, cells were seeded at 5 × 10^3^ cells/cm^2^ in growth media overnight and the medium was replaced with a differentiation medium containing DMEM with 2% FBS, 100 U/mL of penicillin, 100 μg/mL of streptomycin, 2 mM of L-glutamine, 50 μg/mL ascorbic acid 2-phosphate, and 10 mM β-glycerophosphate (Sigma-Aldrich Co., St. Louis, MO, USA). Differentiation media were changed every second day.

### 2.2. Forced Transient Overexpression or Knock-Down of Twist1

For overexpression of Twist1 when OCCM-30 reached 80% confluence in the growth medium, the cells were transfected with Twist1 (kindly donated by Dr. Iseki, Tokyo Medical and Dental School, Tokyo, Japan) cloned to pcDNA4 or pcDNA4 vector alone, using Lipofectamine LTX (Invitrogen, Carlsbad, CA, USA). For knock-down of Twist1, cells were transfected with Twist1 small interference RNA (siRNA) or scramble control siRNA (designed and purchased from Invitrogen), using Lipofectamine RNAiMAX Transfection Reagent according to the manufacturer’s protocol. Transient overexpression or knock-down of Twist1 mRNA expression had been confirmed by Western blotting and the cells were used for subsequent experiments. Each experiment was performed in triplicate as at least three different experiments.

### 2.3. Cell Viability Assays

Cells were plated at a density of 2 × 10^4^ cells/well in 48-well plate. Cells were transfected with plasmid or siRNA at 12 h after cell plating. After 24 h incubation, the CCK-8 (Dojindo Lab, Kumamoto, Japan) solution was added to the culture medium at a final concentration of 0.1 mg/mL, and the cultures were then incubated at 37 °C for 30 min. Subsequently, the absorbance at 450 nm was spectrophotometrically measured using an ELISA reader (BIO Tek Instruments, Winooski, VT, USA). Assays were performed in triplicate for each sample.

### 2.4. Western Blot Analysis

Total proteins were extracted from cells with CytoBuster Protein Extract Reagent (Novagen, Madison, WI, USA). Protein concentration was determined using Amersham GeneQuant Pro (Amersham-Pharmacia Biotech, Arlington Heights, IL, USA). After 50 μg of the proteins were boiled for 5 min in an SDS sample buffer, they were loaded onto 12% SDS-polyacrylamide gel, and the resolved proteins were then transferred to Protran nitrocellulose membrane (Whatman GmbH, Dassel, Germany). The membrane was blocked with 5% skim milk in 10 mM Tris-buffered saline (pH 7.5) plus 0.1% Tween-20 (TBS-T) for 1 h at room temperature with shaking. Then, the membrane was incubated with rabbit polyclonal primary antibody raised against Twist1 (1:1000 dilution with TBS-T containing 5% skim milk) (Abcam, Beverly, MA, USA) and mouse monoclonal primary antibody against β-actin (Sigma-Aldrich) used as the internal control overnight at 4 °C with gentle shaking. The membranes were washed three times in TBS-T to remove nonspecifically bound antibodies and incubated with horseradish peroxidase (HRP)-labeled secondary antibody (1:3000 dilution with TBS-T containing 5% skim milk) (Cell Signaling Technology Inc., Danvers, MA, USA) for 1 h at room temperature. The membranes were again washed three times in TBS-T. Finally, the bound antibodies were reacted with HRP substrate luminol reagent (Millipore Corp., Billerica, MA, USA) and developed bands were photographed using ODYSSEY loaded with Image studio software (LI-COR, Lincoln, NE, USA). Protein bands were quantified by Scion Image software.

### 2.5. ALP Staining

After the cells had been culturing for 3 days in a differentiation medium, the cells were fixed with 3.7% formaldehyde for 10 min. The cells were then rinsed three times with deionized water and BCIP/NBT substrate (Sigma-Aldrich) was added to each well. The enzyme reaction was stopped by the addition of water to each well, and the cells were photographed under a microscope. Stains were quantified by Scion Image software. Three independent experiments were performed in triplicate.

### 2.6. Alizarin Red S Staining

After the cells had been culturing for 6 days in a differentiation medium, they were examined using Alizarin Red S staining to detect calcium depositions. The cells were washed with PBS (phosphate-buffered saline) and then fixed for 30 min with a cold 70% ethanol (Sigma-Aldrich). After the cells were rehydrated with distilled water for 3 min, the calcified nodules were stained with 40 mM alizarin red S solution (Sigma-Aldrich) for 10 min. Cells were rinsed with distilled water twice and photographed. For quantitative analysis, the alizarin red stains were extracted with 10% cetylpyridinium chloride for 15 min. The stains were quantified by measuring the absorbance at 570 nm using an ELISA reader. Each experiment was performed in triplicate as at least three different experiments.

### 2.7. Quantitative Real Time Reverse Transcription Polymerase Chain Reaction (RT)-PCR

Expression of ALP, bone sialoprotein (BSP), dentin matrix protein1 (DMP1), dentin sialophosphoprotein (DSPP), osteopontin (OPN), and runt-related transcription factor 2 (RUNX2) mRNA was assessed by real-time quantitative RT-PCR. The cDNA was synthesized from the total RNA obtained from the experimental and control groups using a with an RT system containing Moloney Murine Leukemia Virus reverse transcriptase (Promega, Madison, WI, USA). Equal amounts of cDNA were used for real-time amplification of the target genes using the SYBR Green PCR Master Mix Reagents Kit (Qiagen, Valencia, CA, USA) running on a Rotor-Gene 3000 (Corbett Research, Mortlake, Australia). The reaction mixture lacking cDNA was used as a negative control in each run. PCR assays were performed in triplicate for each sample to ensure experimental accuracy. PCR conditions were as follow: Incubation for 2 min at 95 °C, followed by 40 cycles of 5 s of denaturation at 95 °C, 15 s for annealing, and extension at 62 °C. PCR Data were analyzed using the Corbett Robotics Rotor-Gene software (version 6.1, Build 90 software). Ratios of the intensities of the target genes and β-actin amplified in internal control were used as a relative measure of the expression level of target genes. The mean fold change of expression in the experimental group, as compared with that in the control group, was calculated using the 2^−ΔΔCt^ method, and the range of the fold changes was calculated from the standard error of the values. Primers were tested for their amplification efficiency and specificity with conventional RT-PCR before being used in quantitative real time PCR. The primer sequences and expected product sizes used are listed in Table 1.

### 2.8. Statistical Analysis

Data were analyzed with Graph Pad Prism^®^ software (version 5.0, Graph Pad Prism, CA, USA). Data are expressed as means ± standard errors. Statistical analysis was performed using ANOVA. Results were considered significant at a probability level of *p* < 0.05. For all experiments, three independent experiments were performed.

## 3. Results

**Figure 1:**
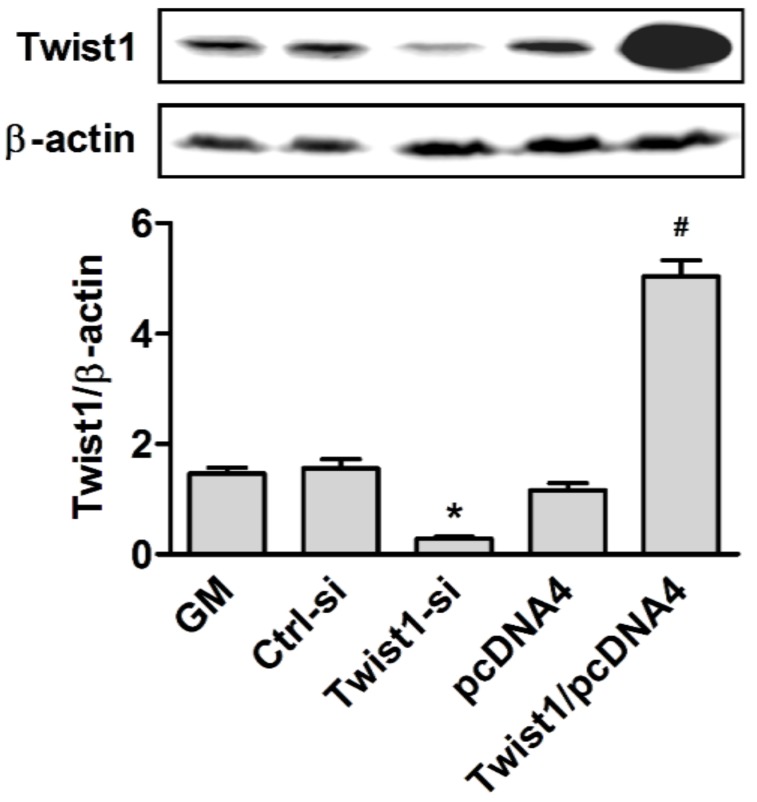
The validation of efficient transfection of Twist1 to cementoblast cells. GM: Growth media. *, *p* < 0.05 compared with the Ctrl-si transfected group; #, *p* < 0.05 compared with the pcDNA4 transfected group.

To investigate the potential role of Twist1 in cementoblast differentiation, OCCM-30 was transiently transfected with Twist1-expressing vectors (Twist1/pcDNA4) to achieve its overexpression or siRNA probes to knock down its expression, using Lipofectamine LTX or Lipofectamine RNAiMAX, respectively. Efficient transfection of Twist1 can be verified by means of Western blot analysis, after incubation for 24 h. Protein bands were showed in upper panel and their intensities quantified by Scion Image software in lower panel (Figure 1). Our results showed successful overexpression of Twist1 (4.3 fold increase, *, *p* < 0.05) compared to the control vector (pcDNA4) by Twist1/pcDNA4, and transient knock down of Twist1 protein (82% decrease, *, *p* < 0.05) compared to the control siRNA (Ctrl-si) by specific siRNA against Twist1 (Twist1-si).

**Figure 2:**
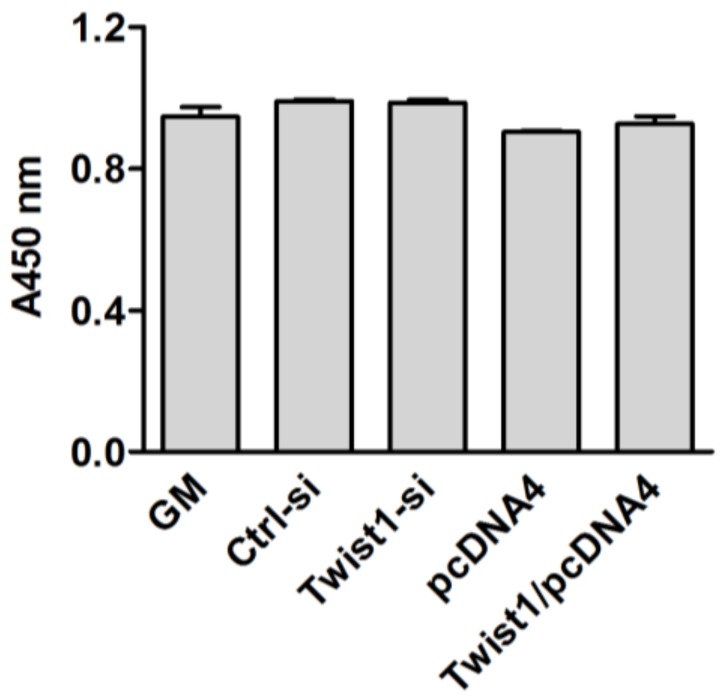
Effect on cellular viability after modulation of Twist1 expression.

To determine whether modulation of Twist1 expression affect cellular viability, cellular viability was measured using the cell counting kit after 24 h of transfection. As shown in Figure 2, forced transfections had no effect on cell viability.

**Figure 3:**
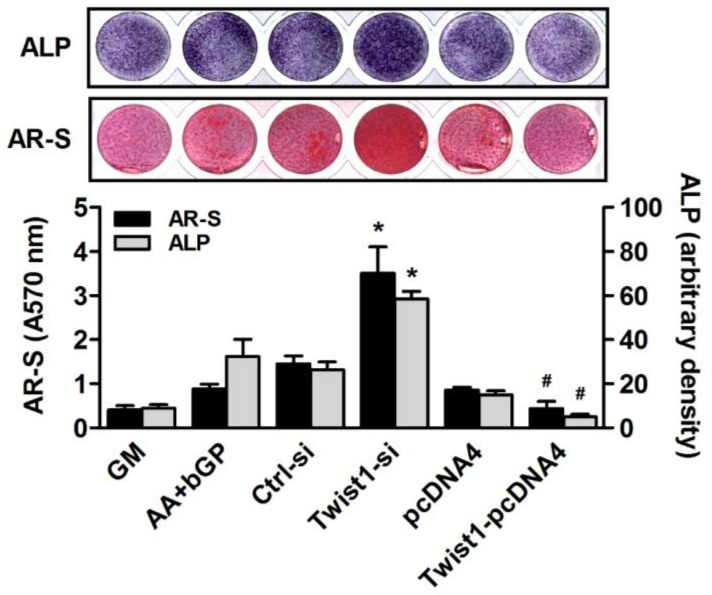
Involvement of Twist1 in differentiation and mineralization of cementoblast cells. *, *p* < 0.05 compared with the Ctrl-si transfected group; #, *p* < 0.05 compared with the pcDNA4 transfected group.

**Figure 4:**
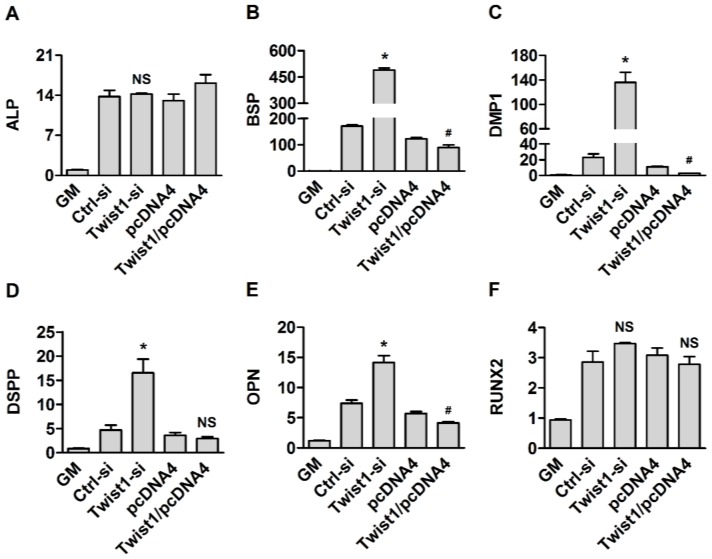
Alteration of cementogenic markers by overexpression or knock-down of Twist1 in cementoblast cells. (**A**) ALP: Alkaline phosphatase; (**B**) BSP: Bone sialoprotein; (**C**) DMP1: Dentin matrix protein dentin; (**D**) DSPP: Sialophosphoprotein; (**E**) OPN: Osteopontin; (**F**) RUNX2: Runt-related transcription factor 2; NS: Not significant. *, *p* < 0.05 compared with the Ctrl-si transfected group; #, *p* < 0.05 compared with the pcDNA4 transfected group.

To assess the effects of transient expression or knock-down of the Twist1 gene on the differentiation potential of cementoblasts, the transfected cells were cultured in the differentiation medium. After 3 and 6 days, ALP and Alizarin Red S stainings were then used to estimate their differentiating capacity and mineral deposition. Densities of alkaline phosphatase stains quantified by Scion Image software and Alizarin Red S stains extracted with 10% cetylpyridinium chloride were shown in lower panel of Figure 3. The Twist1 siRNA-treated group showed significantly higher intensities of both stains than the control (*, *p* < 0.05). As expected, Twist1-pcDNA4-transfected group showed significantly suppressed mineralization abilities of cementoblasts compared with pcDNA4-transfected group (#, *p* < 0.05, Figure 3). Next, qRT-PCR assays were carried out to determine the expression levels of several differentiation markers. Silencing Twist1 upregulated the several cementogenesis markers, such as BSP, OPN, and DMP1 and downregulated expression of ALP, BSP, OPN, and DMP1 with Twist1 overexpression (*, *p* < 0.05). Interestingly, a considerable increase in the expression of DSPP was induced by the knock-down of Twist1. However, little changed in the expressions of ALP and Runx2, other cementogenesis markers by siRNA silencing of Twist1 (Figure 4). 

## 4. Discussion

Our examination of OCCM-30, an immortalized mice cementoblast cell line, revealed that cementoblast differentiation was changed by Twist1 overexpression or knock-down. Unlike virus-mediated methods, transfection approaches using a Lipofectamine could not induce the permanently stable overexpression or silencing of genes, but we were able to verify the changes in protein expressions by means of Western blot analysis. Subsequently, alterations in mineralization capacity and the expression of several cementogenic markers, such as ALP, BSP, DMP1, and OPN, through either overexpression or knock-down of Twist1 would suggest that Twist1 negatively regulated cementoblast differentiation. These findings were in line with the roles of Twist1 in osteoblast differentiation. Unexpectedly, a significant increase in the expression of DSPP by the knock-down of Twist1 was observed in this study. DSPP is an odontoblast-specific marker which has been known to have a very important function in dentin formation [13], and whose expression in the bone and cementum has been demonstrated [14,15]. Recently, its potential functions in cementoblast formation and cementogenesis in vivo and in vitro were suggested as well [16,17]. To reveal connections between Twist1 and DSPP, however, further molecular studies including promotor study will be required.

The mutations in Twist family members, consisting of Twist1 and Twist2 (Dermo-1), are known to be the main cause of Saethre-Chotzen syndrome, which is characterized by craniosynostosis due to premature osteoblast differentiation in the skull and is often associated with facial dysmorphisms and limbs abnormalities [18,19]. Furthermore, dental anomalies, such as teeth with broad, bulbous crowns, thin and narrow tapering roots, and diffuse calcifications in the pulp chamber of posterior teeth, were observed in a patient with this syndrome [20]. A study involving Twist1-deficient mice showed that these animals died shortly after birth, owing to various defects in many major organs due to growth retardation and cachexia [21]. However, other researchers found that Twist1 heterozygous mice survived until adulthood and exhibited calvarial phenotypes similar to craniosynostosis [22,23]. Most investigations that have clarified Twist1 functions have involved in vitro experiments [10,24,25]. Similarly, in our study, we aimed to determine the roles of Twist1 in cementoblast differentiation using the gene gain- or loss-of-function method.

Numerous studies have suggested that Twist1 inhibits osteoblast differentiation by interacting with RUNX2, a transcription factor known to be essential for osteoblast differentiation and osteogenesis [26,27]. Furthermore, based on observations that Twist1-overexpressing cells show de-differentiation or remain in an osteoprogenitor-like state and that antisense-treated Twist1 cells show a differentiated and mature osteoblast-like state, Twist1 is likely to influence osteogenic gene expressions and may function as a master switch initiating bone cell differentiation and thereby regulating the osteogenic cell lineage [28,29]. In this study, the changes of RUNX2 mRNA expression level by both overexpression and knock-down of Twist1 were not observed. Based on this observation, however, it is not to be concluded that Twist1 doesn’t interact with RUNX2 in cementoblast, because, although it is well known Twist1 can suppress transcriptional activity of Runx2, it is still not clear, even in osteoblast, that Twist1 can directly affect Runx2 expression [28,30].

Interestingly, Li et al. [31] reported that Twist1 enhanced dentin-forming odontoblast differentiation from dental stem cells in vitro, which is exact opposite to our findings and previous reports about its roles in osteoblast. This observation was verified by in vivo study showing that stable expressions of Twist1 are essential during odontogenesis [32]. They have suggested that the developmental expression profiles of RUNX2 and Twist1 are different, and so are two transcription factors’ interactions between osteogenesis and odontogenesis. Unfortunately, unlike osteogenesis, cementogenesis cannot be studied as a progressive process owing to the lack of cementoblast-specific markers. Although marker proteins such as cementum-derived attachment protein (CAP) and, more recently, cementum protein 1 (CEMP1) have been reported [33,34], their role still needs to be verified [35,36]. In addition, uncertainty about the developmental origins of cementoblasts might also be considered one of the obstacles to research in this area. Although it is known that the cementoblast originates from periodontal ligament stem cells in adults or from dental follicle cells in developing states, both cells having mesodermal origins, some suggest that it is also derived from the epithelial cells of the tooth organ through epithelial–mesenchymal transformation in developing teeth [37]. Limitations in our experimental approaches cannot clearly explain the discrepancy we found between cementoblast and odontoblast differentiation processes. Therefore, at this moment, it cannot be only said that Twist1 might act in a cell type specific manner. Nonetheless, this study showed that Twist1 does have play a role in regulating cementoblast differentiation. Our results, obtained through gene modifications, might offer a new perspective and strategy for periodontal regeneration. Further studies will be necessary to define the precise molecular interactions between RUNX2 and Twist1 in cementoblasts.

## Figures and Tables

**Table 1:** Sequences of oligonucleotides used for real-time reverse transcription polymerase chain reaction (RT-PCR).

Gene	Primer Sequence	Size (bp)	GenBank Accession No.
ALP	F 5′-TATGGTAACGGGCCTGGCTAC-3′ R 5′-TGCTCATGGACGCCGTGAAGCA-3′	187	NM_007431.2
BSP	F 5′-TGAACAGACTCCGGCGCTAC-3′ R 5′-AGGGCAGCACAGGTCCTAA-3′	127	NM_008318.3
DMP1	F 5′-AGAGGGACAGGCAAATAGTGAC-3′ R 5′-CATTGTCCTCATCGCCAAAGG-3′	176	NM_016779.2
DSPP	F 5′-GGAAGAGCCAAGATCAGGGAA-3′ R 5′-GCCTTGAGGCTTGTCAGACT-3′	173	NM_010080.2
OPN	F 5′-GCCGAGGTGATAGCTTGGCT-3′ R 5′-TGATCAGAGGGCATGCTCAG-3′	177	NM_001204201.1
RUNX2	F 5′-CCAGGCAGGTGCTTCAGAACTG-3′ R 5′-ACATGCCGAGGGACATGCCTGA-3′	157	NM_009820.5
β-actin	F 5′-GATCTGGCACCACACCTTCT-3′ R 5′-GGGGTGTTGAAGGTCTCAAA-3′	138	NM_007393.3
